# Hindlimb muscle spindles inform preparatory forelimb coordination prior to landing in toads

**DOI:** 10.1242/jeb.244629

**Published:** 2023-01-19

**Authors:** Alex Duman, Emanuel Azizi

**Affiliations:** Department of Ecology & Evolutionary Biology, University of California, Irvine, Irvine, CA 92697, USA

**Keywords:** Reinnervation, Spindle Ia afferents, Locomotion, Sensory feedback

## Abstract

Animals move across a wide range of surface conditions in real-world environments to acquire resources and avoid predation. To effectively navigate a variety of surfaces, animals rely on several mechanisms including intrinsic mechanical responses, spinal-level central pattern generators, and neural commands that require sensory feedback. Muscle spindle Ia afferents play a critical role in providing sensory feedback and informing motor control strategies across legged vertebrate locomotion, which is apparent in cases where this sensory input is compromised. Here, we tested the hypothesis that spindle Ia afferents from hindlimb muscles are important for coordinating forelimb landing behavior in the cane toad. We performed bilateral sciatic nerve reinnervations to ablate the stretch reflex from distal hindlimb muscles while allowing for motor neuron recovery. We found that toads significantly delayed the onset and reduced the activation duration of their elbow extensor muscle following spindle Ia afferent ablation in the hindlimbs. However, reinnervated toads achieved similar elbow extension at touchdown to that of their pre-surgery state. Our results suggest that while toads likely tuned the activation timing of forelimb muscles in response to losing Ia afferent sensation from the hindlimbs they were likely able to employ compensatory strategies that allowed them to continue landing effectively with reduced sensory information during take-off. These findings indicate muscle spindle Ia afferents may contribute to tuning complex movements involving multiple limbs.

## Teaser

**Summary:** Toads may rely on hindlimb sensory information during take-off to tune their forelimb muscle activation in order to land effectively.

## INTRODUCTION

Terrestrial locomotion requires animals and humans to navigate complex terrain that may require different control mechanisms to ensure safe and efficient locomotion. Unknown conditions pose the risk of injury, losing out on a meal or being caught by a predator if the individual is unable to coordinate an effective response to that condition. A response to an unexpected perturbation may be coordinated through intrinsic mechanical properties (Daley et al., 2009), or from sensory feedback to alter limb coordination and return the body to steady-state locomotion (Moritz and Farley, 2004). Here, we explored the role of muscle spindle Ia afferents in coordinating safe and efficient locomotion and how the loss of sensory input regarding muscle length may alter movement. By investigating the role of spindle Ia afferents in locomotor control, we can better understand how animals and humans integrate sensory feedback to move effectively.

Peripheral nerve transection and reinnervation has been used as an experimental approach to knock out spindle Ia afferents while allowing complete motor recovery. Peripheral nerve reinnervation is characterized by a loss of sensitivity to muscle length, which is likely caused by a sustained loss of muscle spindle connectivity. Reinnervation causes a significant loss of muscle spindle Ia sensory afferents responsible for the muscle stretch reflex, which persists throughout recovery (Alvarez et al., 2011; Bullinger et al., 2011; Carr et al., 2010; Cope et al., 1994).

Several changes to muscle function occur in response to the reinnervation and subsequent loss of spindle Ia afferents. Immediately following nerve reinnervation, muscle electromyography (EMG) amplitude is significantly reduced and generally does not recover to pre-surgery amplitudes, although it increases throughout recovery in proportion to the reinnervation of endplates at the neuromuscular junction (Gordon and Stein, 1982; Vannucci et al., 2019). Furthermore, Yakovenko et al. (2004) through modeling found the role of Ia (spindle) and Ib (Golgi tendon organ) afferents is dependent on the relative strength of the central control associated with the behavior, with loss of feedback having greater effects on behaviors requiring little central pattern generator (CPG) or central activation, and in severe cases this resulted in the model being unable to support body weight during steady-state locomotion. However, intermuscular feedback from force-sensing Golgi tendon organs remains intact following nerve reinnervation, suggesting that the loss of connectivity of muscle spindles is likely driving the loss of autogenic sensory feedback and the changes in locomotor behavior following reinnervation (Lyle et al., 2016). Pyridoxine-induced Ia afferent ablation also results in reduced joint stiffness in cats and reduced range of motion in the distal hindlimb joints of developing chicks, further suggesting a role of muscle spindle feedback in coordinating proper joint mechanics and indicating a potentially greater reliance on stretch feedback from distal limb muscles in the intact animal (Pearson et al., 2003; Sharp and Bekoff, 2015). Observed changes following nerve reinnervation are thought to be due to a lasting loss of spindle connectivity rather than changes in muscle properties as the electrical properties of spindles remain unchanged and motor units recover tension at a similar rate to whole-muscle tension (Gordon and Stein, 1982; Bullinger et al., 2011).

Several approaches have been shown to result in effective peripheral nerve reinnervation. Methods using laser welding, suturing or gluing all resulted in similar overall functional recovery and recovery rates (Bhatt et al., 2017; Gordon et al., 2020; Gordon and Stein, 1982; Nunes e Silva et al., 2012; Vela et al., 2020). Nunes e Silva et al. (2012) showed nerve-to-nerve (end-to-end or end-to-side) connection is far superior to nerve-to-muscle reinnervation and the use of fibrin or other glues does not significantly improve functional recovery. Most vertebrate models regain locomotor function between 6 weeks and 6 months following reinnervation (Abelew et al., 2000; Bhatt et al., 2017; Carr et al., 2010; Howard et al., 2000; Vannucci et al., 2019). Exercise can be used to accelerate the recovery, with earlier initiation of exercise providing greater recovery than delayed exercise programs (Boeltz et al., 2013; Brandt et al., 2015). Therefore, a simple reinnervation using suture to oppose the cut nerve ends accompanied by an exercise recovery regimen is expected to produce similar results to experiments performed with more intricate surgical procedures.

The loss of muscle spindle Ia afferents resulting from peripheral reinnervation affects some locomotor behaviors more than others. For example, gait characteristics are more significantly affected locomoting downhill compared with walking uphill or level walking because reinnervated muscles are likely undergoing eccentric contractions walking downhill (Abelew et al., 2000; Livingston and Nichols, 2014a,b; Maas et al., 2007). Additionally, the stiffness of joints that reinnervated muscles span is reduced, which is again more apparent in tasks where the muscle is likely lengthened relative to steady-state conditions (Abelew et al., 2000; Chang et al., 2009; Gordon et al., 2020; Livingston and Nichols, 2014a; Maas et al., 2007). Examining the effects of nerve reinnervation in eccentric behaviors where muscles are lengthening and performing negative work is therefore likely to shed more light on the influence of muscle spindle sensory feedback on motor control and coordination.

Frogs and toads have muscle spindles that behave similarly to those of other vertebrates and likely exhibit similar motor control deficits following nerve transection (Jahn, 1968; Proske and Stuart, 1985; Gray, 1957). Most anurans use a saltatory gait where the extension of long powerful hindlimbs propels the animal into the air. As hindlimb muscles are not likely to lengthen during take-off (Azizi and Roberts, 2010), take-off performance is unlikely to be affected by the loss of the muscle stretch reflex. However, this sensory information regarding hindlimb muscle shortening may be important for informing the forelimbs in preparation for landing. While most anuran species have relatively shorter forelimbs (Reynaga et al., 2018), species that specialize in cyclical hopping use their forelimbs to dissipate mechanical energy during landing (Essner et al., 2010). The inability to effectively dissipate the energy associated with a hop can slow locomotor speed and may pose a risk of injury (Nauwelaerts and Aerts, 2006). Terrestrial anuran species that use hopping as a primary mode of locomotion appear to have well-developed and coordinated forelimb behavior to achieve safe and efficient deceleration at landing (Essner et al., 2010; Gillis et al., 2014). Controlled deceleration is achieved through the flexion of forelimbs at impact where eccentric contraction of extensor muscles dissipates the energy (Gillis et al., 2010). Cane toads recruit their elbow extensors earlier during the aerial phase of longer jumps, resulting in a more extended elbow at impact as jump distance increases (Gillis et al., 2010; Azizi and Abbott, 2013; Gillis et al., 2014). This preparatory recruitment which is tuned to the energy that needs to be dissipated at impact is thought to be informed by vestibular and/or hindlimb proprioceptive information during take-off (Cox and Gillis, 2016). However, a direct test of this hypothesis has not been made in freely jumping animals.

In this study, we investigated the role of hindlimb spindle Ia afferents to inform forelimb coordination during landing in the cane toad, *Rhinella marina* (Linnaeus 1758). We use bilateral sciatic nerve reinnervation to ablate the muscle stretch reflex while motor function was regained. Jumping and landing behavior were compared with and without functional spindle Ia afferents. If cane toads rely on sensory input from hindlimb muscle spindles to coordinate forelimb landing behavior and if we successfully ablate the stretch reflex, then we predict: (1) an overall reduction in the rate of elbow extension prior to landing, (2) delayed onset of elbow extension during the aerial phase and (3) reduced elbow extension at touchdown.

## MATERIALS AND METHODS

### Animals

Eleven adult cane toads (6 females and 5 males; body mass: 220.2±85.3 g; snout–vent length: 127.0±14.4 mm; mean±s.d.) were obtained from a commercial supplier and housed in individual terrariums. Toads were fed crickets twice a week and provided with fresh water daily, and the room temperature was maintained between 21 and 22°C on a 12 h light:12 h dark cycle. All research was conducted in accordance with the University of California Irvine's Institutional Animal Care and Use Committee (IACUC) AUP-19-155.

### Stretch reflex test

**Fig. 1.**
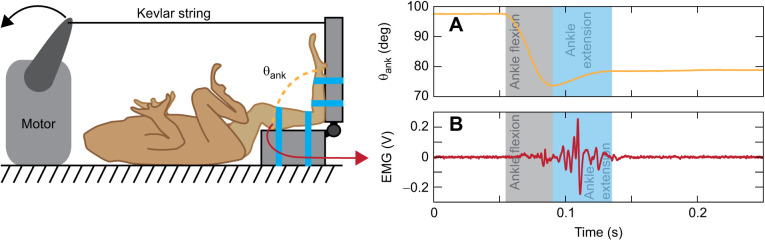
**Muscle stretch reflex experimental setup.** Toads were laid in a supine position with their left hindlimb secured in place to a hinged platform using twist-ties (blue straps in diagram). The hinged apparatus was fixed to the table surface (bottom), which rigidly secured the shank, while the foot was secured to the mobile portion of the apparatus, which allowed the ankle to freely flex and extend. Measurement of (A) the ankle joint angle (θ_ank_) and (B) plantaris muscle activity (electromyography, EMG) was recorded via a bipolar electrode. The gray plot background indicates ankle flexion and the blue background ankle extension; data are from a representative trial of an intact individual prior to any manipulations (pre).

The muscle stretch reflex of the plantaris muscle was tested using a rig that induced rapid ankle flexion and therefore induced rapid plantaris lengthening (Fig. 1). Using high-speed videography at 1964 frames s^−1^, we measured the joint angle throughout and immediately following the perturbation. We used a bipolar electrode (part no. 316SS3T, MedWire Corp, Mt Vernon, NY, USA) inserted into the plantaris muscle to measure the electrical excitation of the motor units within the muscle as the ankle was being rapidly flexed and during the subsequent ankle extension. The stretch reflex was measured in each toad prior to any surgical intervention (*N*=11), after a sham surgery for *N*=4 individuals, in addition to 1 week (*N*=10), 3 months (*N*=9) and 6 months (*N*=8) following the nerve reinnervation surgery for all surviving toads. The stretch reflex was also tested post-mortem (*N*=11) as an additional control for movement artifacts in EMG signals as this would be the only signal recorded in an animal where the nervous system is no longer functioning. Three replicate flexion trials were collected at each of these study time points. One animal died prior to any surgical procedures from unknown causes, another was euthanized for welfare reasons prior to the 3 month time point because of an eye infection that did not respond to treatment, and a third animal died prior to the 6 month time point from an unknown cause.

### Nerve reinnervation procedure

Nerve reinnervation surgeries began with soaking the individual in a chilled bath of 1.5 g l^−1^ MS-222 buffered to pH 7.4 using sodium bicarbonate (NaHCO_3_) for 15–25 min until the animal was unresponsive – determined by a loss of both their righting reflex and toe pinch response. Surgical incisions were made along the ventral surface of each thigh parallel with the femur. Connective tissue was cut and muscle tissue separated to locate the sciatic nerve. Once the sciatic nerve was located for sham surgeries – conducted on *N*=4 toads (2 females and 2 males) – the nerve was left intact, and the skin was sutured (size 4-0) to allow the skin to heal. All other surgeries were conducted by transecting the sciatic nerve proximally to the branching of the tibial and peroneal nerves and sutured (size 6-0) to allow contact between cut nerve ends and facilitate reinnervation. The four individuals that underwent sham surgery recovered for 1 week prior to jumping trials. Following jumping, sham individuals immediately underwent surgery to transect and suture the sciatic nerve as for the other seven toads. After each surgery, toads were injected with 2 mg Carprofen kg^−1^ body mass to reduce inflammation and nociception post-operation. Animals were then monitored closely for the next 48 h for any signs of complication. After 1 week of recovery, each toad was prompted to exercise for 2 min twice a week by walking or jumping at their preferred speed on the hand-powered treadmill to encourage reinnervation and accelerate functional recovery.

### EMG electrode implantation procedure

We used the same anesthetic and preparatory procedures to implant electrodes as we previously detailed for the nerve reinnervation surgery. Our custom bipolar electrodes were constructed from 0.0045 inch (0.1143 mm) diameter insulated stainless steel wire (part no. 316SS3T, MedWire Corp) that was inserted through the dorsal surface of the animal's back and fed under the skin to the anconeus (elbow extensor) and plantaris (ankle extensor) muscles. A ground wire was also submerged subcutaneously on the animal's dorsal side. Following surgery, all toads were injected with 2 mg kg^−1^ body mass Carprofen and allowed to recover for at least 24 h prior to any jump trials.

A custom cable made from 196 cm of 4 channel 22 AWG Shielded cable (part no. 602-2404C-100, Alpha Wire Company, Elizabeth, NJ, USA) was used to connect the toad to the amplifier. All signals recorded from the toads were fed into an A-M Systems Differential AC Amplifier Model 1700 (Sequim, WA, USA), which amplified the muscle signals 1000-fold before recording them into Igor Pro software (WaveMetrics, Portland, OR, USA) via a National Instruments USB-6229 BNC DAQ (16 inputs, 16-bit, 250 kS s^−1^ Multifunctional I/O with Correlated Digital I/O for USB, part no. 197981B-01L, Austin, TX, USA) recording at 10 kHz.

### Jumping procedure

**Fig. 2.**
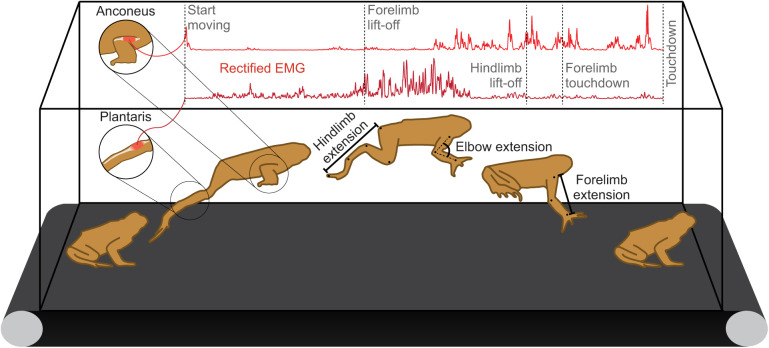
**Continuous jumping experimental setup.** Toads were prompted to jump along a treadmill. Videos were used to track hindlimb and elbow extension throughout each jump and illustrations convey how those values were attained. EMG was recorded from the anconeus and plantaris muscles 6 months following nerve transection and a representative, rectified trace for each muscle is depicted with key times noted along the series.

Toads were placed on a hand-powered treadmill surrounded by Plexiglas walls (43×19×30 cm; l×w×h; Fig. 2) that moved at an approximate speed of 10 cm s^−1^ by the investigator entraining revolutions of the belt to a metronome. The animals were recorded using high-speed SC1 Edgertronic video cameras (San Jose, CA, USA) with Nikon AF Nikkor 50 mm f/1.8D lenses (Minato City, Tokyo, Japan) filming at a rate of 100 frames s^−1^ and angled approximately 30 deg in either direction from the sagittal plane. Videos were taken prior to and after nerve reinnervation surgeries and toads that underwent EMG electrode implantation were filmed at 250 frames s^−1^. Toads were prompted to jump by approaching them from behind with a hand or gently prodding from behind. Filming occurred over 60 s intervals pre-surgery (*N*=10 toads, 200 jumps), 1 week post-sham surgery (*N*=4; 80 jumps), and 6 months post-reinnervation surgery with EMG (*N*=7, 264 jumps). If toads produced fewer than 10 good jumps – defined by the toads not coming into contact with the sides of the jumping arena – then a second 60 s jump trial was recorded during that same day.

### Kinematics

We measured elbow joint and hindlimb kinematics by marking the toads' right side with white out over joints of interest (metatarsophalangeal, ankle, knee, wrist, elbow and shoulder). We applied a dot mark (<2 mm in diameter) with a fine-tip permanent marker (Sharpie) on each joint's center of rotation as determined by moving the joint through its normal range of motion. Our high-speed video was recorded in HD (1080p) giving us 2661 pixels m^−1^ within the jump arena and ensuring the joint center markings were ≤5 pixels in diameter.

In order to quantify hindlimb extension, we also tracked the tip of the right hindlimb toe and we used the bony, posterior protrusion of the ischium as an anatomical landmark for approximating the location of the hip joint. With these markers we were able to estimate and normalize hindlimb extension as the ratio of length between the hip (ischium) and toe tip over the total length of all hindlimb segments. The rate of hindlimb extension was determined by taking the derivative of the hindlimb extension with respect to time. The derivative was calculated by taking the difference in hindlimb extension between the subsequent and previous time point (or video frame) and dividing by twice the time interval between video frames for the pre-surgery and sham surgical conditions when video frame rates were 100 frames s^−1^. The rate of hindlimb extension for post-reinnervation trials (250 frames s^−1^) was calculated by finding the difference in hindlimb extension two frames following and two frames preceding the time point of interest and dividing by four times the interval between video frames to achieve a similar time frame to that used to calculate derivatives for the trials filmed at 100 frames s^−1^. We determined the instantaneous rate of hindlimb extension at take-off as the rate of hindlimb extension on the frame when the hindlimb leaves the ground. Here, a positive value or rate of hindlimb extension suggests the hindlimb is continuing to extend through take-off, while a negative rate of hindlimb extension suggests the toad has begun hindlimb flexion by the time it leaves the ground. The average rate of hindlimb extension was defined by calculating the average of this derivative (rate of hindlimb extension) over the period from the onset of movement until toe lift-off. Rates of elbow extension were calculated in a similar fashion to those for hindlimb extension by seeing how the angle between the wrist, elbow and shoulder changed over time.

### Analysis and statistics

We digitized video data of both the muscle stretch reflex tests and continuous jumping trials using DLTdv8 freeware (Hedrick, 2008). All kinematic measurements as well as EMG analysis were performed with custom MATLAB code. We filtered our continuous jumping kinematic traces using 7th order Savitzky–Golay filters with a frame length of 49 (or length of the vector if it was shorter than 49 frames). We rectified the raw EMG signals from the stretch reflex data and used a 0.1 s window that started 0.15 s prior to any ankle motion to quantify the baseline level of noise and its variation. We set an activation threshold of the average rectified background noise plus two standard deviations (threshold=mean+2 s.d.). We could then quantify the intensity of activity as well as the duration of time the muscle was active (2 s.d. above the average baseline noise) during ankle flexion and extension. The raw EMG signals we recorded from jumping trials were also rectified and then we applied a simple moving average with a window length of 9 samples (approximately 1 ms given our 10 kHz sampling rate). The onset and offset times for both muscles were visually marked for each jumping trial and the intensity was calculated as the area under the rectified trace during the interval of interest.

We employed linear mixed effect (LME) models with individuals treated as a random effect and condition (pre-op, sham, 6 months post-op) as a fixed effect. We compared this model against the null model only containing individuals as a random effect. The AIC value was always lower for the model including experimental condition as a fixed effect. This suggests the model including the condition has greater explanatory power, and therefore we used this model for analyzing each variable. For our EMG data, which we only collected 6 months post-reinnervation, we compared our results with values reported for intact toads in the literature with a one-sample Student's *t*-test. This allowed us to determine whether the timing and duration of activation were significantly different from what others have found in intact cane toads. We used a sequential Bonferroni correction to account for the multiple statistical tests being performed (both LME models and *t*-tests). Any variables that resulted in the condition term of the LME model being significant as determined by the sequential Bonferroni correction (LME, *P*<0.008) were further analyzed using Tukey honest significant difference tests with a critical value of 0.05.

## RESULTS

**Fig. 3.**
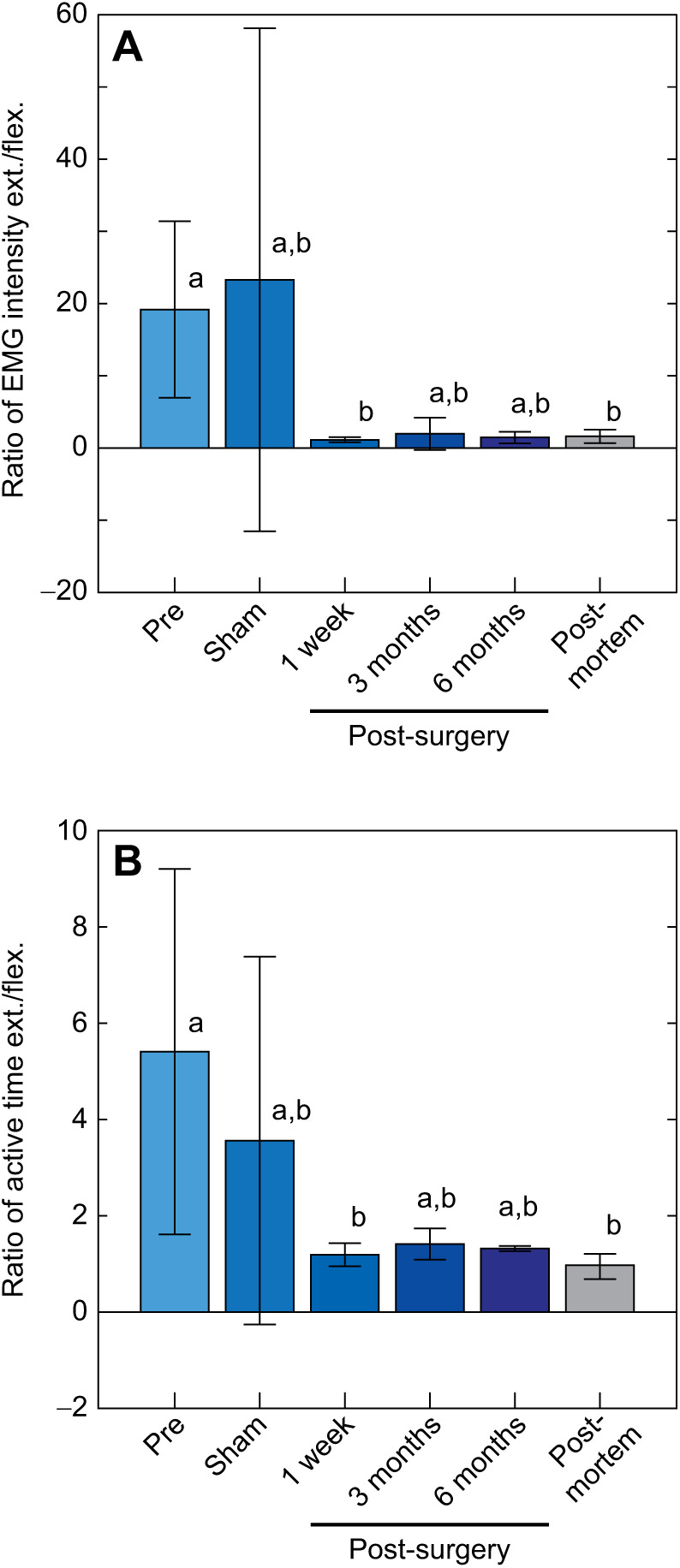
**Measuring the muscle stretch reflex before and after surgery and during recovery.** Data are shown for intact toads before surgery (Pre; *N=*11), after sham surgery (Sham; *N*=4), at 1 week (*N*=10), 3 months (*N*=9) and 6 months (*N*=8) following nerve reinnervation, as well as post-mortem (*N*=11). (A) The ratio of EMG intensity (measured in V s) occurring during ankle extension relative to the intensity occurring during ankle flexion (ext./flex.), and (B) the ratio of time (measured in s) the plantaris is active during ankle extension relative to time the plantaris is active during ankle flexion. Bars represent condition means, error bars convey the 95% confidence interval (CI) of the mean, and different lowercase letters above bars signify a significant difference between condition means.

We tested for the presence of the muscle stretch reflex prior to surgery, 1 week following the sham surgery, and throughout recovery from the nerve transection surgery to illustrate the sustained loss of the muscle stretch reflex (Fig. 3). The ratio of plantaris EMG intensity during ankle extension relative to ankle flexion significantly decreased from pre-surgery (*N=*11) to 1 week post-reinnervation (*N*=10) as well as post-mortem (*N*=11; Fig. 3A; LME, *F=*4.94, *P*=0.001). We found that the pre-surgery ratio of EMG intensity did not meet the *a priori* level of significance for the 3 month post-reinnervation (*N*=9, Tukey HSD, *P*=0.060) or 6 month post-reinnervation (*N*=8, Tukey HSD, *P*=0.061). Similarly, the sham condition did not meet the significance criteria for any post-reinnervation or post-mortem conditions; however, all Tukey HSD *P*-values were less than 0.10. All time points following the nerve reinnervation surgery, including post-mortem, had average ratios near one (range: 1.16–1.98) with all their 95% confidence intervals (CI) for the mean encompassing a ratio of one which would be expected from random noise during both phases of ankle motion. We observed a similar pattern for the ratio of EMG duration in the plantaris during ankle extension relative to flexion (Fig. 3B; LME, *F=*3.66, *P*=0.007). Again, the stretch reflex of the intact animal activated the plantaris significantly longer during extension than during flexion as compared with the 1 week post-reinnervation and post-mortem time points. Comparisons between the intact animals did not meet the criteria for significance 3 months (Tukey HSD, *P*=0.070) or 6 months (Tukey HSD, *P*=0.075) following reinnervation surgery. While the average ratio of activation duration following the sham surgery was greater than at all time points following reinnervation surgery, the difference was not significant. These data suggest we ablated or at the very least significantly reduced the muscle stretch reflex through our nerve transection surgeries throughout the course of the 6 month recovery period.

**Fig. 4.**
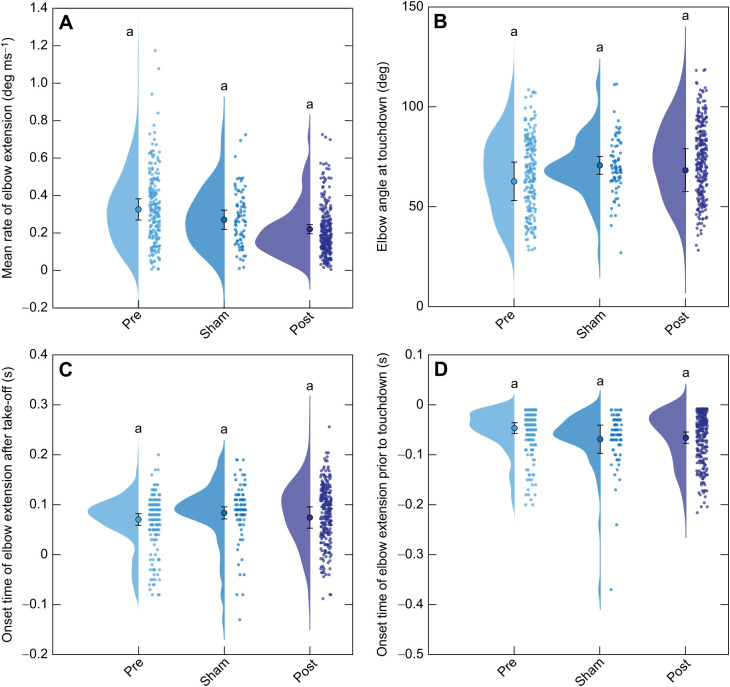
**Forelimb coordination before surgery and during recovery.** Data are shown for toads before surgery (Pre; *N*=10, 200 jumps), after sham surgery (Sham; *N*=4, 80 jumps) and 6 months following nerve reinnervation (Post; *N*=7, 264 jumps). (A) The mean rate of elbow extension, (B) the elbow angle at touchdown, (C) the time following hindlimb take-off when the elbow first extends, and (D) the time prior to touchdown when the elbow first extends during the aerial phase. Clouds and small circles to the right of the same color represent the distribution and all jumps recorded for that condition, respectively. The larger central circle represents the condition mean after accounting for individual variation and error bars convey the 95% CI of the mean. Different lowercase letters above distributions represent statistically significant differences in the mean.

We observed no significant differences in forelimb kinematics in preparation for landing (Fig. 4). The reduction in mean rate of elbow extension during the aerial phase 6 months following nerve reinnervation (0.220±0.024 deg ms^−1^; mean±95% CI of the mean, henceforth referred to as 95% CI_μ_ for brevity) relative to the intact condition was not significant (0.326±0.057 deg ms^−1^, mean±95% CI_μ_; Table 1, Fig. 4A; LME, *F=*5.41, *P*=0.014). The toads also had similar elbow extension at touchdown in both the sham condition (70.7±4.4 deg, mean±95% CI_μ_) and 6 months post-reinnervation (68.3±10.7 deg, mean±95% CI_μ_) compared with the intact condition (62.7±9.7 deg, mean±95% CI_μ_; Fig. 4B; LME, *F=*2.67, *P*=0.096). We also found that the onset time of elbow extension following take-off was not significantly different between intact, sham and 6 month post-surgery conditions (Fig. 4C; LME, *F=*1.25, *P*=0.310). Additionally, the onset time of elbow extension prior to touchdown was not significantly different between any of the conditions analyzed (Fig. 4D; LME, *F=*3.40, *P*=0.056). These data fail to provide support for our initial hypotheses that (1) the rate of elbow extension would be reduced, (2) the onset of elbow extension would be delayed, and (3) elbow extension at touchdown would be reduced following hindlimb reinnervation.

**Table 1.**
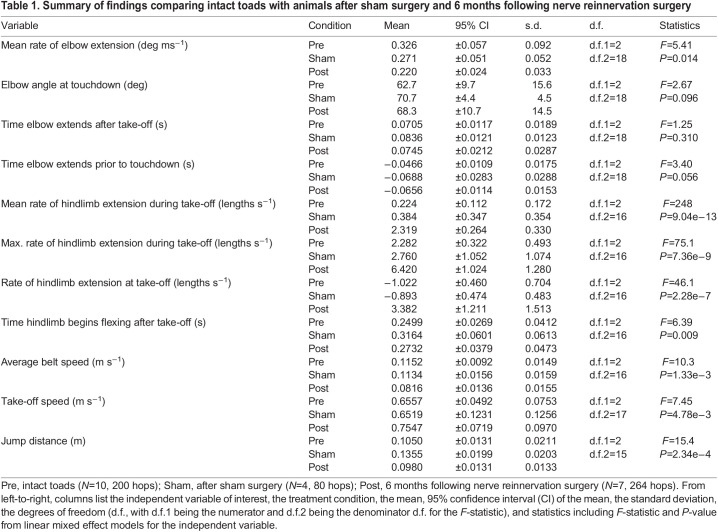
Summary of findings comparing intact toads with animals after sham surgery and 6 months following nerve reinnervation surgery

**Fig. 5.**
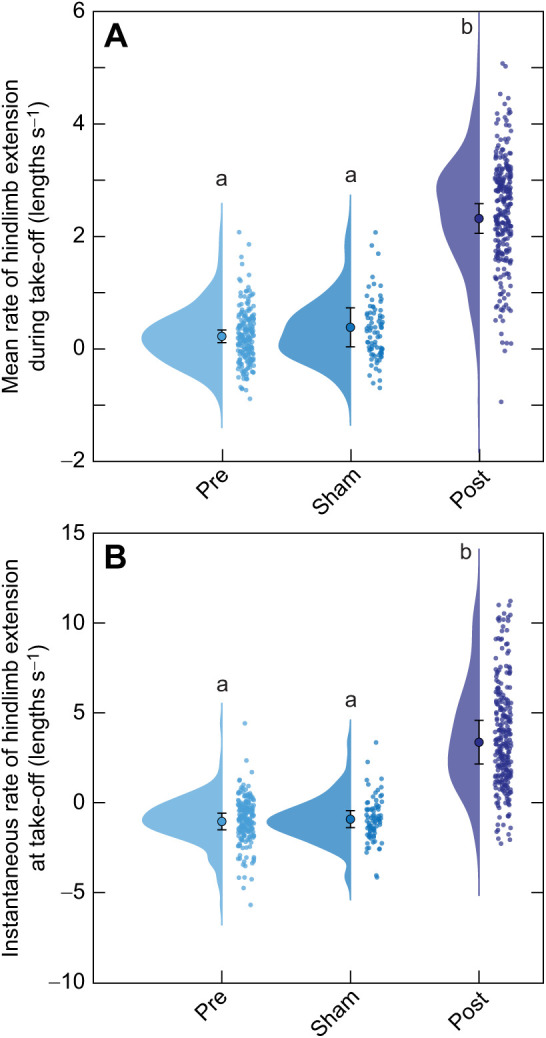
**Hindlimb coordination before surgery and during recovery.** Data are shown for toads before surgery (Pre; *N*=10, 200 jumps), after sham surgery (Sham; *N*=4, 80 jumps) and 6 months following reinnervation (Post; *N*=7, 264 jumps). (A) The mean rate of hindlimb extension during take-off as measured from the onset of motion to toe lift-off, and (B) the instantaneous rate of hindlimb extension at the time of hindlimb take-off. Clouds and small circles to the right of the same color represent the distribution and all jumps recorded for that condition, respectively. The larger central circle represents the condition mean after accounting for individual variation and error bars convey the 95% CI of the mean. Different lowercase letters above distributions represent statistically significant differences in the mean.

We also quantified hindlimb kinematics before and after surgery because performing nerve reinnervation surgery in the proximal hindlimb was invasive as much of the connective tissue had to be cut and muscle displaced to reach and sever the sciatic nerve. We found that while the average rate of hindlimb extension during take-off did not significantly differ between the intact (0.224±0.112 lengths s^−1^, mean±95% CI_μ_) and sham (0.384±0.347 lengths s^−1^, mean±95% CI_μ_) conditions, it did significantly increase 6 months post-reinnervation (2.319±0.264 lengths s^−1^, mean±95% CI_μ_; Table 1, Fig. 5A; LME, *F=*248, *F=*1.70, *P*<0.0001). We found a similar trend for the maximal rate of hindlimb extension during take-off, with pre-surgery (2.28±0.32 lengths s^−1^, mean±95% CI_μ_) and sham (2.76±1.05 lengths s^−1^, mean±95% CI_μ_) conditions having significantly lower maximal hindlimb extension rates compared with that of toads 6 months following nerve reinnervation (6.42±1.02 lengths s^−1^, mean±95% CI_μ_; LME, *F=*75.1, *P*<0.0001). Furthermore, the instantaneous rate of hindlimb extension at take-off was significantly increased 6 months after reinnervation (3.38±1.21 lengths s^−1^; mean±95% CI_μ_) compared with both the intact (−1.02±0.46 lengths s^−1^, mean±95% CI_μ_) and sham surgical conditions (−0.89±0.47 lengths s^−1^, mean±95% CI_μ_; Fig. 5B; LME, *F=*46.1, *P*<0.0001). Although we report specific shifts in hindlimb performance that likely affected take-off performance (e.g. take-off speed), the overall jumping behavior (e.g. jump distance) we observed from these animals remained largely similar 6 months following nerve transection surgery compared with trials on intact animals (Table 1; see also Fig. S1).

We additionally measured the EMG activity of the anconeus and plantaris muscles 6 months following nerve reinnervation surgery. We found the plantaris – the primary ankle extensor – initially became active 0.216±0.066 s (mean±95% CI_μ_) prior to take-off. The anconeus – the primary elbow extensor – had an activation duration of 0.0291±0.0127 s (mean±95% CI_μ_) during the aerial phase, and initially became active −0.0035±0.0030 s (mean±95% CI_μ_) prior to hindlimb lift-off.

## DISCUSSION

### Forelimb behavior

While we observed a minor reduction in the mean rate of elbow extension in preparation for landing following sciatic nerve reinnervation, the trend was not significant and suggests reinnervated toads extend their elbow at rates similar to intact conditions. This result does not support our initial prediction and the findings of Cox et al. (2018) regarding the reduced rate of elbow extension following sciatic nerve transection. Toads initiated elbow extension at a similar time relative to both take-off and touchdown following nerve reinnervation as compared with their intact state, which also fails to support our prediction of a delayed onset of elbow extension. Sciatic nerve transection may immediately alter forelimb landing behavior in the cane toad, although it also inhibits motor control of the hindlimbs and normal take-off behavior, resulting in the toads being unable to initiate jumps voluntarily (Cox et al., 2018). Because our experimental manipulation allowed for motor recovery while maintaining a sustained loss of the muscle stretch reflex in the distal hindlimb, our results suggest feedback from muscle spindles in the hindlimb is not entirely necessary for coordinating effective forelimb-mediated landing behavior.

**Fig. 6.**
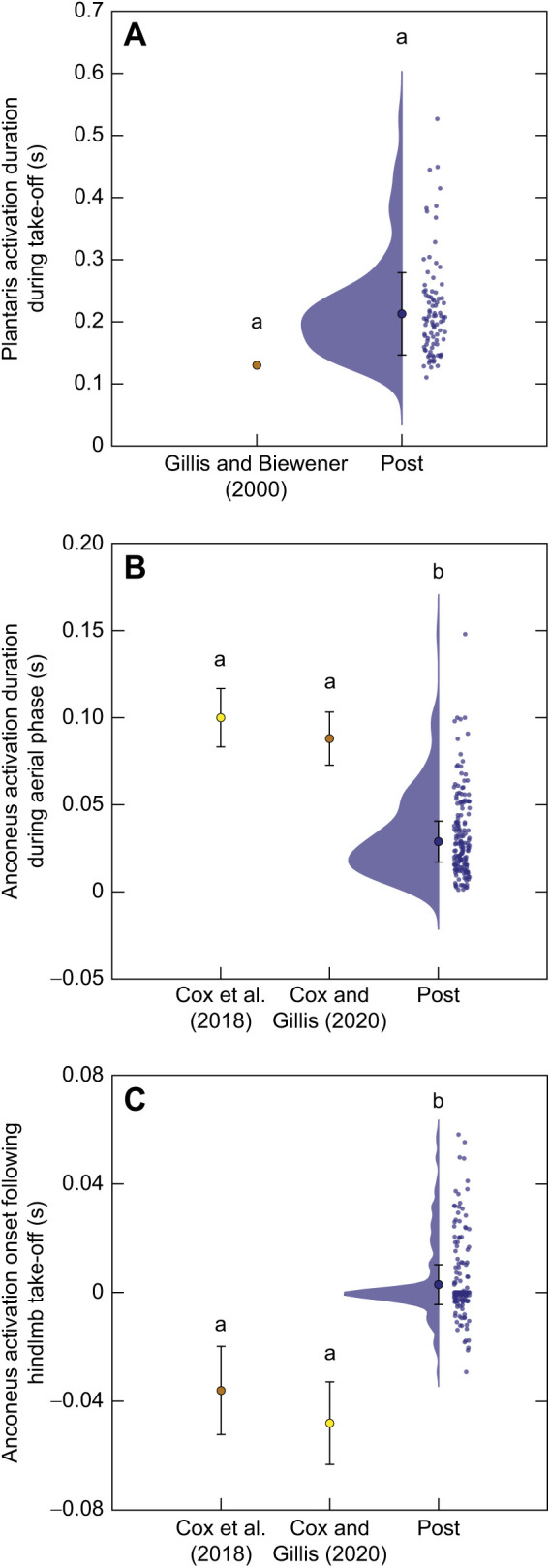
**Muscle activity 6 months following nerve reinnervation (post).** (A) The duration of plantaris (*N*=6; 91 jumps) activity prior to take-off, (B) the duration of anconeus (*N*=6; 187 jumps) activation during the aerial phase, and (C) the onset time of the anconeus (*N*=6; 187 jumps) following hindlimb take-off. Data are from the present study, Gillis and Biewener (2000), Cox et al. (2018) and Cox and Gillis (2020). Clouds and small circles to the right of the same color represent the distribution and all jumps recorded, respectively. The larger central circle represents the condition mean after accounting for variation within an individual and error bars convey the 95% CI of the mean. Different lowercase letters above datasets represent statistically significant differences in the mean (one sample *t*-test, *P*<0.008).

We also showed a significant reduction in the duration of anconeus (elbow extensor) activation during the aerial phase in toads 6 months post-reinnervation compared with the intact toads from Cox et al. (2018) (one-sample *t*-test, *P*<0.0001) and Cox and Gillis (2020) (one-sample *t*-test, *P*<0.0001; Fig. 6B). Additionally, the onset of anconeus activation during the aerial phase in the post-reinnervation condition was significantly delayed – occurring closer to hindlimb take-off – compared with values reported in Cox and Gillis (2020) (one-sample *t*-test, *P*<0.0001) and from Cox et al. (2018) (one-sample *t*-test, *P*<0.0001; Fig. 6C). Our findings suggest muscle spindle Ia afferents from the distal hindlimb influence the onset timing and duration of anconeus (elbow extensor) activation, resulting in the muscle activating later and for a shorter duration during the aerial phase of the jump. While we report significant changes in the timing of EMG activity of the anconeus, we did not observe significant changes in the kinematics of the elbow in preparation for landing. This suggests that the animals may have developed compensatory strategies to overcome a delayed onset of activation and overall shorter duration of activation during the preparatory aerial phase. One potential mechanism could be an increase in the magnitude of muscle activation to account for shorter activation times, producing similar elbow angles at touchdown; however, we are unable to test this hypothesis given the difficulty normalizing EMG intensity between different animals and that we were limited to comparisons of intact toads from pre-existing literature.

Elbow extension determines the orientation of the two longest forelimb segments, and therefore maintaining relatively similar elbow extension kinematics likely equates to similar overall limb extension between intact and reinnervated states. Toads may have altered the activation of forelimb muscles following nerve reinnervation but are likely attempting to conserve limb-level and center of mass dynamics at impact. This aligns with research showing that cats conserved their hip kinematics and limb contact angles following reinnervation in distal limb muscles (Abelew et al., 2000; Chang et al., 2009; Maas et al., 2007). Chang et al. (2009) reported greater flexion of the distal joints (another common effect of reinnervation) in cats that was compensated for by greater extension in the hip. Additionally, we only report on a single time point after relatively complete recovery of motor function. Chevallier et al. (2004) found that salamanders following spinal transection initially exhibit increased variability in step duration and forelimb–hindlimb coupling during walking, which both gradually converged back to the intact condition over time. This re-development of intact behavior may explain why we did not observe a significant change in elbow mechanics 6 months following surgery because the toads had sufficiently recovered and converged back to their intact landing behavior. While we only report here on data collected 6 months following reinnervation, Chevallier et al. (2004) suggest we might expect the kinematic data collected 3 months following surgery (not reported here) to have greater variation as muscles reinnervate and neural circuits are potentially in the process of reorganization to accommodate a loss of hindlimb spindle Ia afferents.

Upstream neural reorganization within the central nervous system likely occurs following nerve damage. Spinal transections result in the regeneration of neural networks that restores behavior but these networks are anatomically and functionally reorganized from their intact state (Haspel et al., 2021; Parker, 2017). While our sciatic nerve transection in the hindlimbs is not as severe an alteration to the structure of the central nervous system as a spinal transection, our finding of a shift in the activation onset and duration of the anconeus suggests potential upstream changes to the circuits coordinating landing behavior or that these neural circuits mediating preparatory forelimb landing behavior can be modulated via spindle Ia afferents coming from the hindlimb. Frogs share motor synergies (the coactivation of several muscles that leads to a characteristic movement) between swimming and jumping behaviors to simplify control across movement modalities (Cheung et al., 2005). Therefore, we hypothesize that our reinnervation may affect one or more of the motor synergies associated with the take-off and landing phase of a jump. Because the central nervous circuitry – likely in the spinal cord – is cut off from spindle Ia afferents in muscles distal to the reinnervation site during take-off, the circuitry may be reorganized or at the very least alter its descending motor commands to effectively coordinate a landing with the forelimbs in the absence of muscle length information from the whole hindlimb.

### Hindlimb behavior

In addition to assessing forelimb mechanics, we showed the average rate of hindlimb extension was significantly greater following nerve reinnervation than what we observed in both the intact and sham conditions. We additionally found that the instantaneous rate of hindlimb extension at take-off was significantly higher in the reinnervated animal compared with that in both the intact and sham conditions, suggesting that the reinnervated animal continues to extend its hindlimb through take-off. This is not the case for intact or sham toads, which appear to initiate flexion earlier in the aerial phase. We observed this same trend for the maximum rate of hindlimb extension, and noted that toads had a more difficult time keeping their feet and toes planted on the substrate following reinnervation. This slipping of the feet could be a reason for the greater rate of hindlimb extension observed in the reinnervated condition. Because we only observed a significant increase in the rate of hindlimb extension during take-off following reinnervation surgeries and not in the sham condition, it is likely caused by the loss of muscle spindle sensory information. Cutting connective tissue in the thigh during surgery that normally serves to maintain muscle shape and transfer force during take-off, and may be essential for both jump propulsion and hindlimb retraction after take-off, risks affecting normal jump performance (Astley and Roberts, 2012, 2014; Reynaga et al., 2019; Ruttiman et al., 2019; Schnyer et al., 2014). Our results indicate that any disruption of the mechanical properties of the hindlimb musculature were relatively minor and likely did not cause the observed shifts in hindlimb extension following reinnervation because the sham group did not exhibit this deviation in behavior from the intact state. We did not investigate the hindlimb extension at touchdown, although hindlimb extension at touchdown could have implications for the toad's ability to coordinate an effective landing as they have been shown to alter hindlimb retraction to effectively minimize torques on their forelimbs upon impact (Azizi et al., 2014).

EMG was recorded in the plantaris (primary ankle extensor) muscles during jumping in the toads at 6 months following their nerve reinnervation surgery. We compared our findings on the activation timing of the plantaris muscle with results reported for the same muscle in cane toads from Gillis and Biewener (2000). We found that the plantaris muscle was active for a duration approximately 50% longer on average during take-off following sciatic reinnervation compared with that in intact animals; however, these results were not considered significant following the sequential Bonferroni correction (one-sample *t*-test, *P*=0.023; Fig. 6A). Given that our hindlimb kinematics were significantly altered by reinnervation, it is likely the activity of the plantaris muscle may also have been shifted. Gordon et al. (2020) reported a shift toward earlier activation of reinnervated lateral gastrocnemius muscle and greater shortening velocity at peak force generation compared with that of the intact muscle. Toad hindlimbs are in a relatively ineffective mechanical position during take-off and initiating movement from the ankle may therefore demand a greater duration of plantaris activation following reinnervation if the contraction velocity necessary to produce similar work is increased (Reynaga et al., 2019).

### Limitations

We recognize our comparison of the sham group 1 week following surgery and the reinnervated group 6 months following surgery is not ideal. However, to reduce the number of individuals needed and refine our methods while also minimizing pain and distress in accordance with IACUC, we opted to record the sham group jumping after 1 week of recovery. We also ideally would have recorded muscle activity from the intact and sham conditions. But leaving subcutaneous electrodes embedded for extended periods of time increases the risk of infection and significantly reduces quality of life and so we decided to only measure muscle activity in reinnervated animals and compare it with pre-existing values from the literature. This does not detract from the significant differences we report between intact and reinnervated animals and only applies to comparisons between our sham and reinnervated conditions.

While the duration of plantaris activation during take-off was not significantly different following reinnervation, we observed changes in the rate and timing of hindlimb extension during take-off, which suggests we did alter hindlimb mechanics by reinnervating the sciatic nerve. We cannot rule out that muscles distal to the transection site were cross-reinnervated – motor neurons reinnervated different muscles from those innervated originally before transection (Abelew et al., 2000). Cross-reinnervation of motor units limits our ability to draw accurate comparisons between reinnervated and intact animals if the motor commands sent to specific muscles in the hindlimb are significantly altered from the intact state.

Another limitation due to the location of our sciatic nerve transection being relatively distal is that connections to proximal thigh musculature were left intact. Cheung et al. (2005) suggest the semitendinosus and other hamstring muscles are important for providing feedback for modulating motor synergies in frogs. Our method of reinnervating the distal sciatic nerve leaves the neural feedback from these thigh muscles intact. Again, this limitation does not take away from the significant changes in anconeus activation we observed due to proprioceptive ablation in distal hindlimb muscles.

### Conclusion

Through bilateral reinnervation of the sciatic nerve in the hindlimb of cane toads, we successfully ablated the muscle stretch reflex distal to the transection site. We also observed a shift toward later activation and shorter overall duration of activity in an elbow extensor muscle while elbow kinematics were conserved. This suggests the muscular activation for coordinating jumps in our reinnervated toads was tuned to preserve forelimb preparatory behavior and land effectively. Losing muscle spindle sensory information from the distal hindlimb is not sufficient to fundamentally alter landing behavior, but it may play a role in tuning and timing forelimb muscle activation in preparation for landing. However, the mechanism responsible for the changes we observed is still unknown. Future efforts should investigate ablating spindle Ia afferents from all hindlimb musculature using similar techniques and track how the nervous system potentially compensates for a more complete loss of muscle spindle feedback. This can be achieved by tracking changes in the morphology and connectivity within the reinnervated nerve as well as the spinal cord via neuroimaging, MRI or fluorescence labeling (Cizkova et al., 2020; Haspel et al., 2021; Seif et al., 2019). Additionally, tracking changes in motor synergy structures following peripheral nerve reinnervation may provide insight into how animals alter activation of limb muscles following the loss of spindle Ia afferent sensory information.

## Supplementary Material

10.1242/jexbio.244629_sup1 Supplementary information
