# Supplementary material for: Hindlimb muscle spindles inform preparatory forelimb coordination prior to landing in toads
Source: J Exp Biol. 2023 Jan 19;226(2):jeb244629. doi: 10.1242/jeb.244629 (PMC10086541; doi:10.1242/jeb.244629)
Supplement: Supplementary information [file jexbio-226-244629-s1.pdf]

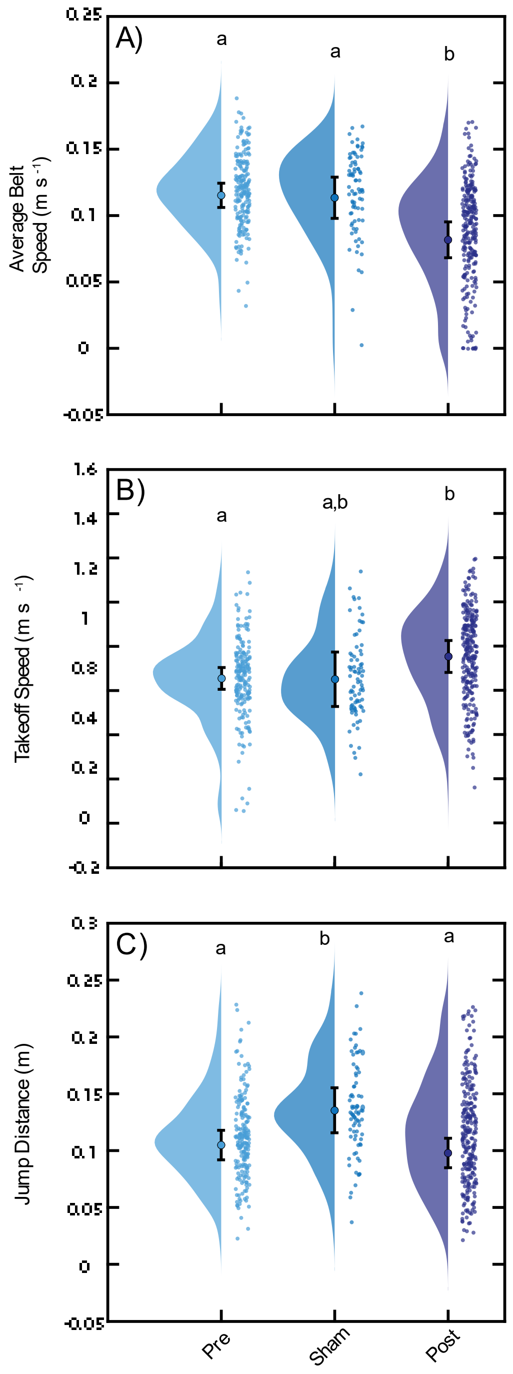

Fig. S1. Jump conditions before surgery (pre; *N* = 10, 200 jumps), after sham surgery (sham; *N* = 4, 80 jumps) and 6 months following nerve reinnervation (post; *N* = 7, 264 jumps). A) The average treadmill belt speed during the jump, B) instantaneous speed of COM at time of hindlimb takeoff, and C) jump distance. Clouds and small points to the right with the same color represent the distribution and all jumps recorded for that condition, respectively. The larger central circle represents the condition mean after accounting for individual variation and error bars convey the 95% CI of the mean. Lowercase letters above distributions that differ represent statistically significant differences in the mean.

Journal of Experimental Biology • Supplementary information
